# Aortic valve replacement in a young patient with essential thrombocytosis

**DOI:** 10.1186/1749-8090-3-5

**Published:** 2008-01-30

**Authors:** Kashif Ahmed, Hunaid A Vohra, Alison Milne, Stephen M Langley

**Affiliations:** 1 Department of Cardiothoracic Surgery, Southampton University Hospitals NHS Trust, Southampton, UK; 2 Department of Haematology, Basingstoke & North Hampshire Foundation Trust Hospital, Basingstoke, UK

## Abstract

Essential Thrombocythcythaemia (ET) is an uncommon type of myeloproliferative disorder, characterised by both thrombotic and haemorrhagic diathesis. No clear guidelines exist for the pre- and post-operative management of patients undergoing cardiac surgery in the haematological and surgical literature. This condition has profound implications in patients undergoing cardiac surgery with the use of cardiopulmonary bypass, where heparin is used for anti-coagulation. This dilemma is further compounded in the setting of a young patient undergoing aortic valve replacement (AVR), where insertion of a mechanical prosthesis would be the procedure of choice. This would require life-long anticoagulation with warfarin which can predispose these patients to catastrophic bleeding. Using a tissue valve will subject the patient to multiple redo operations in the patient's lifetime. We report a young patient with ET requiring AVR and discuss the dilemmas surrounding the choice of prosthesis in this patient.

## Case report

**Figure 1:**
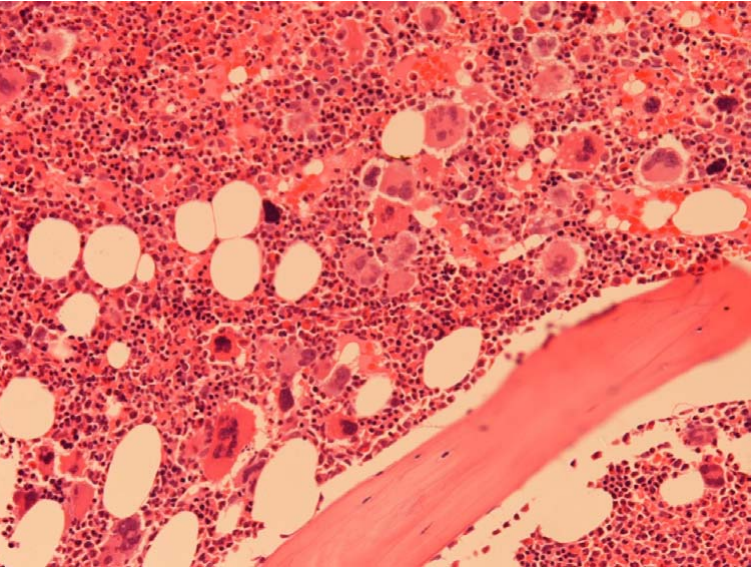
Histology of the bone marrow trephine biopsy in our patient showing increased number of megakaryocytes (Haematoxylin & Eosin staining, Magnification 20×).

A 22 year old gentleman was referred to our cardiothoracic centre for consideration for aortic valve replacement (AVR). He was diagnosed with moderate aortic valve stenosis at birth. The aortic valve was bicuspid and there was no associated significant left ventricular hypertrophy as a neonate. He had no other past significant medical history except for migraine. He was asymptomatic. At the age of 5 years the peak gradient (PG) across the aortic valve was 30 mmHg on trans-thoracic echocardiogram (TTE). At follow-up, 1 year before referral, there was an increase in the PG to 50 mm Hg followed by an increase to 86 mm Hg two weeks before referral. The patient denied any symptoms. At the age of 21, he was found to have an isolated thromocytosis with a platelet count of 874 × 10^9^/L with a normal haemoglobin, haematocrit and white blood cell count. His inflammatory markers were negative. The platelet count was repeatedly above 800 × 10^9^/L. There was no history of bleeding or thrombocytosis and no splenomegaly on examination. He was referred to a haematologist. A bone marrow aspirate showed increased number of megakaryocytes. The bone marrow trephine showed classical Essential Thrombocythaemia (ET) (figure 1). Cytogenetics were normal. Blood was negative for JAK-2 gene. In view of his age (<40 yrs), absence of hypertension and diabetes, he was categorised as low risk and started on aspirin alone. However, the planned aortic valve surgery necessitated controlling the platelet count over the perioperative period to reduce his risk of bleeding or thrombosis. The options were short-term use of hydroxycarbamide with a theoretical leukaemia and teratogenic risk or α-interferon. The patient elected to have α-interferon. Within three months the platelet count was normal, 301 × 10^9^/L prior to surgery. He underwent an uncomplicated AVR with a 23 mm mechanical prosthesis (Carbomedics Inc, Texas, USA) using a semi-continuous technique with 2/0 prolene suture. Cardiopulmonary bypass time was 30 minutes and the cross-clamp time was 19 minutes. One million units of trasylol was administered in the CPB circuit and another one million in the patient. The total bleeding in the first 24 hours was 500 mls. The post-operative recovery was uneventful without any complications. He was anti-coagulated with intravenous heparin 24 hours after surgery to maintain an activated plasma thromboplastin ratio (APTR) between 2.5–3.0. At the same time oral aspirin and warfarin were commenced and once the international normalised ratio (INR) was between 2.5–3.0, heparin was stopped, he was discharged home on day 5 post-op. Platelets on discharge were 369 × 10^9^/L and Hb 110 g/L.

## Discussion

The pathophysiology of ET is still uncertain after more than 70 years of its first description. It was described by Epstein and Goedel in 1934 [1], as a non-reactive, chronic myeloproliferative disorder. This condition is characterised by sustained megakaryocyte proliferation in the bone marrow which increases the number of circulating platelets in the blood. The cause of this increase in platelet production remains unclear, though it may be a result of autonomous production, increased sensitivities to cytokines (eg, interleukin-3), decreased inhibition to platelet-inhibiting factors (eg, transforming growth factor beta), or defects in accessory cell microenvironment [2]. Platelet survival is, however, normal. Traditionally, ET was considered a clonal disorder that involved pluripotent stem cells; however, recent studies indicate that some patients may have polyclonal haematopoiesis [3]. Classically, ET leads to splenomegaly and follows a clinical course complicated by haemorrhagic and/or thrombotic episodes.

During cardiopulmonary bypass (CPB), there is activation of platelets by thrombin (mainly), contact with non-endothelial cells, heparin (administered during CPB), and platelet-activating factor produced by a variety of cells during extracorporeal perfusion and re-circulation of anti-coagulated blood that has been exposed to a wound. Platelets aggregate with each other and to monocytes and neutrophils. This, along with the large procoagulant surface of the circuit overwhelming natural circulating anticoagulants (antithrombin, protein C, protein S, tissue factor pathway inhibitor and plasmin) producing thrombin may lead to thrombosis within the circuit. The factors affecting thrombin generation during CPB include the amount and type of anticoagulant, surface area of the blood-biomaterial interface, duration of exposure, turbulence, stagnation, cavitation within the circuit, temperature and characteristics of the circuit [4]. That the wound is the major source of thrombin generation during CPB has encouraged development of strategies to discard wound blood [5] or salvage red cells by centrifugation in a cell saver. The mechanism by which thrombocytosis produces thrombosis is not well defined. Several defects have been described including hyperaggregation of platelets and intra-platelet concentration of various chemicals. Some reports show patients with an acquired deficiency of antithrombin III, protein C, and protein S. Thrombosis may be serious and life threatening. Especially, thrombosis in the CPB circuit during cardiac surgery may prove to be catastrophic resulting in interruption of extra-corporeal circulation, vital organ malperfusion and thrombo-embolic complications. The obvious complex interplay of factors affecting platelet function in patients with ET undergoing cardiac surgery with CPB has not been studied and merits future research.

Cardiac surgery also predisposes the patient to a bleeding tendency post-operatively. This is mainly due to fibrinolysis caused by blood contact with the CPB circuit and by blood suctioned from the operative field. Platelet dysfunction, heparin effect, haemodilution (reduced platelets) and hypothermia (during CPB and post-operatively) also contribute to postoperative bleeding. Circulating platelets express GPIb and GPIIb/IIIa receptors [6], and the platelet mass consists of a reduced number of morphologically normal platelets, some abnormally shaped platelets, new platelets, degranulated platelets, platelet membrane fragments and microparticles [7]. Due to the above, bleeding times increase and remain prolonged for several hours after protamine. The phenomenon of 'heparin rebound' despite complete neutralization with protamine occurs frequently. Heparin increases the sensitivity of platelets to soluble agonists, inhibits binding to von Willebrand factor, and modestly increases template bleeding times. Reports have shown that in patients with ET, there is a decrease in von Willebrand ristocetin cofactor activity and high molecular weight von Willebrand factor multimers [8]. The results of the prothrombin time and activated partial thromboplastin time studies are usually within reference ranges, but the bleeding time may be prolonged. In ET, the site of bleeding is usually the gastrointestinal tract but is mild in most cases. In view of the factors discussed above, this could transform into serious haemorrhage in ET patients who have undergone cardiac surgery recently. In an experimental setting, aprotinin has been shown to promote platelet adhesion [9] as well as decrease postoperative bleeding in patients undergoing cardiac surgery with platelet dysfunction (treated with clopidogrel <5 days before surgery) [10]. It is for this reason we used aprotinin in our patient.

A further dilemma in the setting of a young ET patient undergoing aortic valve replacement (AVR) is what type of prosthesis to use. Normally, a mechanical valve would avoid the risk of structural deterioration that occurs with tissue prostheses but would require life-long anticoagulation with warfarin which can predispose ET patients to catastrophic bleeding. Using a tissue valve or homograft will subject the patient to multiple redo operations in the patient's lifetime which would subject the patient to thrombo-embolic and bleeding risks of cardiac surgery alluded to above. In our case, detailed discussions were carried out with the haematologist. The patient was considered a low-risk ET patient due to being young with no previous history of bleeding, no hypertension or diabetes a young age and good response to treatment. In consultation with the patient it was decided that life-long warfarin would be a safe option and a mechanical valve was inserted.

The issue of haemorrhagic diathesis would become even more serious in the rare instance of transformation of ET into AML [11] in patients on warfarin, in which case more aggressive treatment (chemotherapy and bone marrow transplantation) may be required. Stopping anti-coagulation in the event of bleeding complications (mainly bleeding gums, purpura and multiple ecchymoses) or for therapeutic intervention (allogenic or autologous bone marrow transplantation) may lead to valve thrombosis and eventual mechanical valve failure. Bleeding may be caused by thrombocytopenia, coagulopathy that results from disseminated intravascular coagulation (DIC), or both.

Management of ET patients undergoing cardiac surgery under CPB should be individualized based on risk factors for thrombo-haemorrhagic complications. Patients undergoing surgery are at increased risk for bleeding and thrombosis. Advice from haematologist must be sought to help manage patients with ET and monitor therapy. Observation may be appropriate for low-risk patients. High risk patients include older patients (>60 years), history of thrombosis, platelet count >1500 m/ml, which is paradoxically associated with an increased risk of GI tract bleeding in young women, obesity, cardiovascular risk factors and markers of hypercoagulability such as factor V Leiden and antiphospholipid antibodies [12]. Recommend lifestyle modifications (eg, weight loss for obese patients, smoking cessation for smokers). One should strongly consider administering cytoreductive drugs like hydroxycarbamide, anagrelide, interferon α to reduce the platelet count to the reference range prior to surgery [13]. In addition, low-dose aspirin may be useful in the prevention microvascular occlusion and major bleeding. In an emergency, plateletpheresis may be useful to achieve a rapid decrease in platelet counts in the setting of acute thrombosis and/or marked thrombocytosis. Splenectomy performed for any reason can markedly increase the platelet count and the risk of both hemorrhagic and thrombotic events.

## Conclusion

The decision to choose a mechanical versus tissue prosthesis in young ET patients requiring AVR is not an easy one. Until more research reveals laboratory investigations and/or markers which can clearly define patients more prone either to bleeding or thrombosis, clinical strategy will need to be tailored on the basis of clinical presentation of ET and weighing the risks and benefits of the treatment options available.

## Competing interests

The author(s) declare that they have no competing interests.

## Authors' contributions

KA and HV were involved in the writing of the report while AM and SML corrected and finalised the manuscript from the haematology and surgery point of view, respectively. All authors read and approved the final manuscript.
